# Interdependence of Nutrition, Physical Activity, and Myopia

**DOI:** 10.3390/nu16193331

**Published:** 2024-09-30

**Authors:** Małgorzata Mrugacz, Katarzyna Zorena, Magdalena Pony-Uram, Maja Lendzioszek, Kamila Pieńczykowska, Anna Bryl

**Affiliations:** 1 Department of Ophthalmology and Eye Rehabilitation, Medical University of Bialystok, 15-089 Bialystok, Poland; malgorzata.mrugacz@umb.edu.pl; 2 Department of Immunobiology and Environmental Microbiology, Medical University of Gdansk, 80-211 Gdansk, Poland; katarzyna.zorena@gumed.edu.pl; 3 Department of Ophthalmology, Subcarpathian Hospital in Krosno, Korczynska 57, 38-400 Krosno, Poland; magdalena.ponyuram@gmail.com; 4 Department of Ophthalmology, Voivodship Hospital in Lomza, 18-400 Lomza, Poland; lendzioszek.majka@gmail.com; 5 Clinical Hospital, Medical University of Bialystok, 15-089 Białystok, Poland; mynameiskama@gmail.com

**Keywords:** myopia, diet, lifestyle, myopia prevention, myopia complications

## Abstract

**Background:** Myopia (also known as nearsightedness), a prevalent refractive error, occurs when parallel rays of light converge in front of the retina, resulting in blurry distance vision. Recently, there has been a marked rise in myopia among the global population. The absence of effective methods of controlling the progression of this visual defect prompts the search for new preventive and therapeutic options. The impact of diet and lifestyle on the progression of myopia is still not fully understood. Therefore, our aim was to examine how these factors might affect the advancement of myopia, based on the existing literature. **Methods:** This manuscript was prepared through an extensive literature review conducted from June 2022 to September 2024. We searched for pertinent research articles using reputable databases, including PubMed, Scopus, and Web of Science. We included all types of publications, with a special focus on the newest ones. **Results:** Despite far-reaching examination, the relationship between these factors and myopia control remains inconclusive with varying degrees of evidence supporting their roles. **Conclusions:** However, promoting a healthy lifestyle, particularly increasing physical activity and outdoor time, is essential. Additionally, emerging research suggests that maintaining a balanced diet is important due to the potential impact of certain nutrients on myopia development. Ophthalmologists should also guide parents on the alternative correction methods beyond single vision glasses, especially for rapidly progressing cases. With the rising prevalence of myopia in children, further research is necessary.

## 1. Introduction

Myopia constitutes a refractive error in which parallel rays of light are focused in front of the retina. This results in a lack of clarity in distant vision. In 2010, it was estimated that myopia and high myopia affected 27% (1893 million) and 2.8% (170 million) of the global population, respectively [1]. In 2020, approximately 34% of the world’s population was suffering from myopia [2]. Predictions indicate that by 2050, half of the world’s population will be affected by myopia [2,3]. It has been established that the occurrence of myopia may be influenced by both genetic [1,4] and environmental factors, of which close work is mentioned the most often [5]. Over the past generation, the incidence of myopia has rapidly increased worldwide [3]. In East Asia, estimates suggest that up to 90% of the population may experience myopia, with 20% potentially developing high myopia [6,7]. According to China News, the prevalence of myopia among children and adolescents in China averaged 53.6%. A significant upward trend in the defect was observed with age. The prevalence of myopia in China was 36.0% among primary school students, was 71.6% among lower secondary school students and, among high school students, it was as much as 81.0% [8]. An increase in the incidence of myopia has also been observed in Europe and the USA [9,10]. The research group on the prevalence of eye diseases estimated the prevalence of myopia to be 26.6% in Europe, 25.4% in North America, and 16.4% in Australia [10]. In individuals of European ancestry, there are the following six loci linked to refractive error: FAM150B-ACP1, LINC00340, FBN1, DIS3L-MAP2K1, ARID2-SNAT1, and SLC14A2; in Asian populations, the following three genome-wide significant loci have shown strong interactions with education: AREG, GABRR1, and PDE10A, [1]. People from different Hispanic backgrounds and Mexican Americans have an elevated risk of developing nearsightedness, which may be linked to their genetic background. The degree of myopia was found to have a positive correlation with height, due to a relationship between height growth and axial length increase in children and adolescents [11]. The repercussions of myopia are known to contribute to substantial social and economic issues. In their work, Naidoo et al. reported that the global productivity lost due to uncorrected myopia was estimated at USD 244 billion annually. In Singapore, the annual direct cost of optical correction of myopia in adults was estimated at USD 755 million [12]. During the COVID-19 pandemic, remote work, both among students and adults, along with the surge in multimedia development, significantly contributed to the upswing in myopia [13,14]. Unfortunately, no standardized prophylactic or therapeutic procedure that could prevent the development and progression of this visual defect exists. It is believed that the application of atropine drops to the conjunctival sac may be effective in limiting the progression. Unfortunately, this method is not free from side effects. Mydriasis and the associated photophobia, loss of accommodation, diminishing long-term efficacy, and rebound effects are significant limitations [15,16]. Therefore, the lack of an effective way to inhibit the development and progression of myopia leads to the search for new therapeutic options. Our understanding of the influence of diet and lifestyle on myopia’s progression remains circumscribed. In our research, we undertook the task of scrutinizing the prevailing scientific literature concerning the impact of diet and lifestyle on myopia’s development and progression.

## 2. Materials and Methods

**Figure 1:**
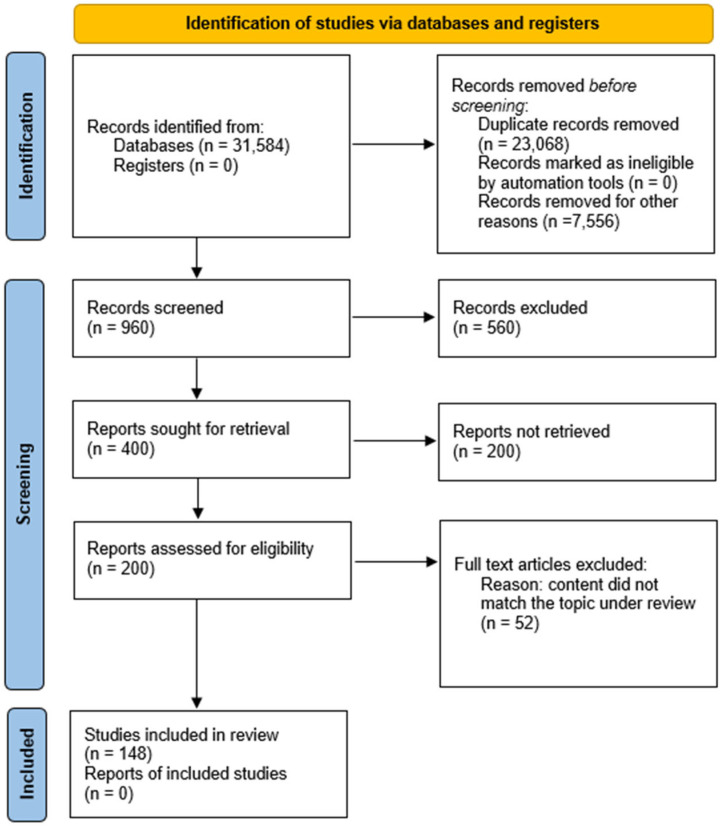
PRISMA flowchart of study selection process.

This systematic review was conducted and reported based on the preferred reporting items for systematic reviews and meta-analyses (PRISMA) statement and the PRISMA network meta-analysis extension statement (Supplementary Materials) [17]. The preparation of this manuscript involved a comprehensive literature review starting from June 2022 to September 2024. We utilized reputable databases, such as PubMed, Scopus and Web of Science, to search for the relevant research articles. The purpose of this review paper was to evaluate the recent research that explores the relationship between diet, lifestyle, and the influence of these factors on the development and progression of myopia. Therefore, our search terms comprised various combinations of the following keywords and phrases: “myopia”, “diet and the development of myopia”, “lifestyle and the development of myopia”, “diet and the progression of myopia”, “lifestyle and the progression of myopia”, “myopia prevention”, “myopia complications”, “myopia control”, and “myopia health”. Only publications in English were selected. The geographical location of the studies was not a determining inclusion factor. In addition, our inclusion criteria focused on recent publications, with a particular emphasis on publications from the past five years, unless the papers presented new and groundbreaking insights or mechanisms. We considered all types of studies. Exclusion criteria were applied to studies that were not accessible in their entirety online or published in other languages than English. Studies were also excluded if they were conference abstracts or posters, or if they did not pertain to the research question. Ultimately, we selected 155 articles from 1958 to 2024 to include in this review (shown in Figure 1).

## 3. Myopia and Its Consequences

As previously mentioned, myopia ensues when parallel light rays converge anteriorly to the retina, causing the distant vision to blur. In addition to cases of genetic myopia [18], close work and reading are thought to cause hyperopic defocus through a weak accommodative response to nearby objects [19,20,21]. Optical blurring resulting from delayed accommodation may therefore be a stimulus that contributes to excessive ocular elongation [19,20,21]. The prevalence of myopia ranges from 3% among schoolchildren in the Sub-Saharan African countries to approximately 85% among older high school students in parts of East and Southeast Asia [22,23,24]. Studies have also established that the female gender constitutes an added risk factor for myopic development [22,25,26]. Compared to boys, girls’ sleep duration has a stronger indirect impact on myopia through outdoor activities and BMI. In boys, the mediating effect of sleep duration on myopia through outdoor activities was 25.0%, whereas in girls, it was 32.65%, which was notably higher than in boys. As for the relationship between sleep duration and myopia, among boys BMI mediates only 5.65%, while in girls, the mediation effect is higher at 12.10%. This gap is likely mainly due to hormonal changes during puberty. Girls experience puberty earlier than boys and undergo more pronounced changes in height, weight, and BMI, making them more susceptible to the effects of myopia [27]. Girls tend to be less active outdoors while spending more time engaged in reading and writing compared to boys. Another possible reason of this differences might be that elevated estrogen levels during adolescence can alter the shape of the eye [28]. A higher occurrence of myopia was found in the children living in cities (31.1%) than in the children living in rural areas (18.0%) [29,30]. Williams et al. found in their study that myopia was more than twice as common in people with higher education [31]. Children of both myopic parents had a 4.8 times higher risk of myopia compared to children of parents without myopia [32]. It has been found that the earlier myopia develops in a child, the higher the risk of high myopia in adulthood [33]. Rosner et al. found no relationship between myopia and body height or body mass index in a study conducted on a large group of men aged 17–19 [34]. Complications of myopia are a consequence of the elongation of the eyeball. They include sclera and choroid thinning, as well as retinal thinning that may result in the development of degenerative changes and an increased risk of retinal detachment [35]. Disturbances in the blood supply to the structures of the eyeball, which accompany its elongation, are also significant [36,37,38]. High myopia predisposes an individual to subfoveal choroidal membrane incidence [35,39] and increases the risk of damage to the optic nerve in the course of increased intraocular pressure [35], which may result in the irreversible loss of vision. Haarman et al. reported that myopic people have a 100 times higher risk of myopic macular degeneration, a three times higher risk of retinal detachment, a three times higher risk of posterior subcapsular cataract, and almost twice as high a risk of open-angle glaucoma [40]. The incidence of myopic macular degeneration ranged from 0.1% to 7%, from 0.3% to 10%, and from 13% to 65% in low, moderate, and high myopia, respectively [41,42,43]. There are several theories attempting to explain the higher risk of developing cataracts in nearsighted people. Myopic eyes may be exposed to higher levels of oxidative stress or lower levels of glutathione in the lens, which may accelerate the development of cataracts. Second, higher levels of lipid peroxidation by-products in myopia may also accelerate cataract formation. Also, a longer eyeball may lead to reduced diffusion of nutrients from the vitreous chamber to the lens [40]. Doshi et al. found that a longer axial length leads to optic disc tilt and secondary damage to the axons in the lamina cribrosa, which results in the development of glaucoma [44]. Some authors also describe an increased risk of myopia in people with diabetes [45,46]. However, it should be noted that diabetes may contribute to an increased risk of developing nuclear cataracts [47]. In their work, Du et al. discussed many metabolic mechanisms underlying myopia [48]. It is believed that nitric oxide may play an important role. Nitric oxide (NO) is a messenger that plays a key role in oxidative stress. NO is recognized for facilitating light-adaptive changes in the retina, with its production and release being elevated in response to intense or intermittent light exposure [49]. Vultipongsatorn et al. showed that lowering oxidative stress helps reduce the progression of myopia by suppressing chronic scleritis [50]. In vivo, NO is mainly synthesized by nitric oxide synthase (NOS). NO activates guanylate cyclase (cGMPase). Dysregulation of the NO–cGMP signaling pathway can cause various diseases, including myopia [51]. The interaction between gallic acid and nitric oxide is beneficial in various diseases that involve inflammation and oxidative stress. Gallic acid (GA) is a polyphenolic compound found in various plants, fruits (e.g., bananas, pineapples, lemons or strawberries), and teas. GA treatment prevented the degradation of endothelial nitric oxide synthase (eNOS) and maintained NO levels. Moreover, inhibiting eNOS activity with a specific inhibitor (L-NG-nitroarginine methyl ester) significantly eliminated the beneficial effects of GA [52]. Lai et al. discovered that the inclusion of the antioxidant GA in the structure of functionalized gelatin-g-poly(*N*-isopropylacrylamide), which was created to enhance the therapeutic efficacy of intracamerally administered pilocarpine, can improve the cytoprotective properties of carrier materials against oxidative stress caused by hydrogen peroxide in the lens epithelial cell cultures [53]. Fujikado et al. effectively inhibited myopia in chickens by injecting Nω-nitro-L-arginine methyl ester (L-NAME), a NOS-specific inhibitor, directly into the vitreous. This reduced the expression of NOS in the retina and regulated the occurrence of myopia [54].

## 4. Myopia Correction Methods and Their Impact on the Defect Progression

Numerous methods are available to correct myopia, with those most commonly employed to slow its progression, encompassing progressive lenses, Defocus Incorporated Multiple Segments (DIMS) spectacle lenses, monofocal and multifocal contact lenses, orthokeratology, pharmacological agents, and nonpharmacological therapy [19,55,56,57]. In a study spanning two years, Malinowski et al. observed the progression of myopia in 102 patients aged 8 to 20 years, stratified by the correction method used, including the following: single vision spectacle lenses, single vision contact lenses (SVCL), and multifocal contact lenses (MFCL—Appendix + 2.00). They observed the smallest progression of myopia in the group of people corrected with the MFCL [19]. Holden et al. obtained similar results [57]. In addition, Malinowski et al. observed the greatest inhibition of myopia by the MFCL in the initial period of the study, which would suggest a beneficial start of therapy with this type of correction. In the second year of follow-up, the difference in myopia progression between the SVCL and MFCL groups decreased and was no longer statistically significant. After two years of observation, they found a statistically significant difference in the progression of the defect between the groups using the MFCL and single vision glasses in favor of the MFCL, but they did not find a statistically significant difference between the groups using the SVCL and the single vision glasses, and between the MFCL and the SVCL. Notably, when dealing with myopia following a period of intense growth, no disparities in defect progression were evident based on the correction method [19]. Walline et al., in a study encompassing children aged 8–11 years, showed that the progression of myopia was as follows: −1.03 ± 0.06 diopters (D) with the SVCL and −0.56 ± 0.06 D with the MFCL. A similar increase in the axial length of the eyeball was smaller with the use of the MFCL [20]. The COMET-2 study showed, over a 3-year observation period, that progressive contact lenses with the addition of +2.0 D slowed down the progression of myopia by 24% [58]. High hopes are associated with the Defocus Incorporated Multiple Segment (DIMS) eyeglass lens [56]. DIMS comprises a central optical zone for myopia correction, surrounded by multiple segments inducing constant blur (+3.50 D) [56,59]. Children wearing DIMS spectacle lenses exhibited 52% less myopia progression and 62% less axial elongation of the eye compared to children wearing monofocal spectacle lenses [59]. Orthokeratology is employed to correct low and medium myopia. Ortho-K lenses are rigid contact lenses worn at night, whose task is to flatten the cornea [56]. Their operation is based on the hypothesis of myopic blurring of the peripheral retina. The effectiveness of Ortho-K in inhibiting the progression of myopia is estimated from 32% to 63% [39,60,61]. Cho et al. compared the progression of myopia in children aged 7–12 using orthokeratology and monofocal spectacle lenses [60]. In the group using orthokeratology, the average increase in the axial length of the eyeball was 0.29 mm and was significantly lower than in the group of children using single vision spectacle lenses (0.54 mm) [60]. Similar results were obtained by Kakita et al. [62]. During a five-year follow-up, the axial eye length increased by 0.99 mm for children using orthokeratology and 1.41 mm for those using the single vision glasses, with the most significant treatment effect observed in the first year. The axial length differences between the two groups were significant in the first, second, and third years of follow-up but not in the subsequent two years. Notably, the side effects of orthokeratology use, as described in the literature, primarily pertain to conjunctival and corneal inflammation, often resulting from inadequate care and not exhibiting a significantly higher occurrence rate than with soft contact lens users [63]. Walline et al., in a study involving 40 children aged 8–11, demonstrated the superiority of orthokeratology over monofocal soft contact lenses, with the axial eye length increasing by 0.25 mm over two years in the orthocorrection group and 0.57 mm in monofocal soft contact lens wearers [64].

Li et al. conducted an analysis of atropine’s impact on myopia progression in children aged 4 to 12. They found that younger patients exhibited a weaker response to atropine treatment, necessitating higher atropine concentrations to achieve results similar to older children. Over a 2-year period, 6-year-old children receiving 0.05% atropine showed a mean progression of −0.90 D, comparable to the 8-year-olds receiving 0.025% atropine (−0.89 D). In the 10-year-olds, a concentration of 0.01% atropine was sufficient for a progression of −0.92 D. Importantly, all age groups tolerated these concentrations well [65]. Yen et al. assessed the effectiveness of 1% atropine and 1% cyclopentolate eye drops. They observed that the myopia progression with atropine was −0.22 ± 0.54 D/year, whereas with cyclopentolate, it was −0.58 ± 0.49 D/year. In the control group using placebo eye drops, myopia progression was the highest at −0.91 ± 0.58 D/year [66]. The ATOM study reported that the axial length of the eyeball remained stable in individuals using 1% atropine drops (−0.02 ± 0.35 mm/2 years), in contrast to the study group experiencing an axial elongation of 0.38 ± 0.38 mm/2 years [67]. In the ATOM2 study, the impact of lower concentrations of atropine eye drops on myopia progression was assessed. Myopia progression was −0.30 ± 0.60 diopters with 0.5% atropine, −0.38 ± 0.60 diopters with 0.1%, and −0.49 ± 0.63 diopters with 0.01%. The axial elongation of the eyeballs was 0.27 ± 0.25 mm, 0.28 ± 0.28 mm, and 0.41 ± 0.32 mm, respectively [16]. Side effects of topical atropine application included mydriasis, reduced accommodation, photophobia, and dry mouth [68,69,70]. A rebound effect was observed upon discontinuation of the drops, with a greater rebound effect associated with higher concentrations of the drops used in treatment. According to Chia et al., one year after the treatment’s end, myopia progressed by −0.87 ± 0.52 D with 0.5% atropine, −0.68 ± 0.45 D with 0.1%, and −0.28 ± 0.33 D at the 0.01% concentration of atropine used [71]. Based on the ATOM-2 study results, the topical application of 0.01% atropine is recommended for medical prophylaxis of myopia progression [72]. It is worth noting that full or partial myopia correction or the use of monovision did not appear to impact myopia progression [35,73,74].

## 5. Effect of Diet on the Progression of Myopia

Studies examining the influence of diet on myopic development have not provided unequivocal findings. Yin et al. stated that maintaining a diet consisting of a high intake of meat, seafood, dairy products, eggs, legumes, vegetables, fruits, grains, and potatoes has a protective effect when it comes to myopia [75]. Subjects with myopia generally have higher total body fat percentage, body mass index (BMI), or waist circumference. Across different age groups, the variations in fat percentage based on refractive error show significance in individuals under 40 years old but not in those aged 40 and above [76]. The relationship between myopia and an elevated BMI was confirmed by Qu et al. [77]. Lee et al., for instance, identified a connection between childhood and adolescent obesity and the occurrence of high myopia, particularly among girls. Their research demonstrated that obese children and adolescents have a 3.77 times greater risk of developing high myopia than their normal-weight peers. This risk was even more pronounced among girls, with a 5.04 times higher likelihood compared to boys, where it was 2.84 times higher [78]. Similar conclusions have been reached by other researchers [79,80]. The exact mechanism underlying the link between obesity and myopia remains unclear, but insulin resistance has emerged as a significant factor. This condition is present in 15% to 20% of obese children [78,81]. Increased consumption of foods with a high glycemic index is believed to promote insulin resistance, especially among female patients [82,83]. Elevated insulin levels in the bloodstream trigger the secretion of insulin-like growth factor 1 (IGF-1), which influences cell growth and differentiation, ultimately leading to axial elongation of the eyeball and the development of myopia [78,82,84]. Haarb et al. also found a correlation between insulin levels and myopia [3]. In individuals without diabetes, hyperglycemia can suppress insulin secretion, resulting in lens thickening and an anterior shift of the anterior pole, which exacerbates myopia [78,85]. On the other hand, consuming large amounts of fruit may help lower the risk of myopia, as fruits contain phytochemicals (such as carotenoids) that can prevent the condition [83]. According to Brown et al., axial myopia results from increased fluid retention in the vitreous of the eye, triggered by dietary sodium chloride consumption. Salt enhances the ionic permeability of retinal membranes and increases the osmotic gradient, causing fluid to flow into the vitreous. This process stretches the ocular tissue during axial elongation [86].

### 5.1. Macronutrients

**Figure 2:**
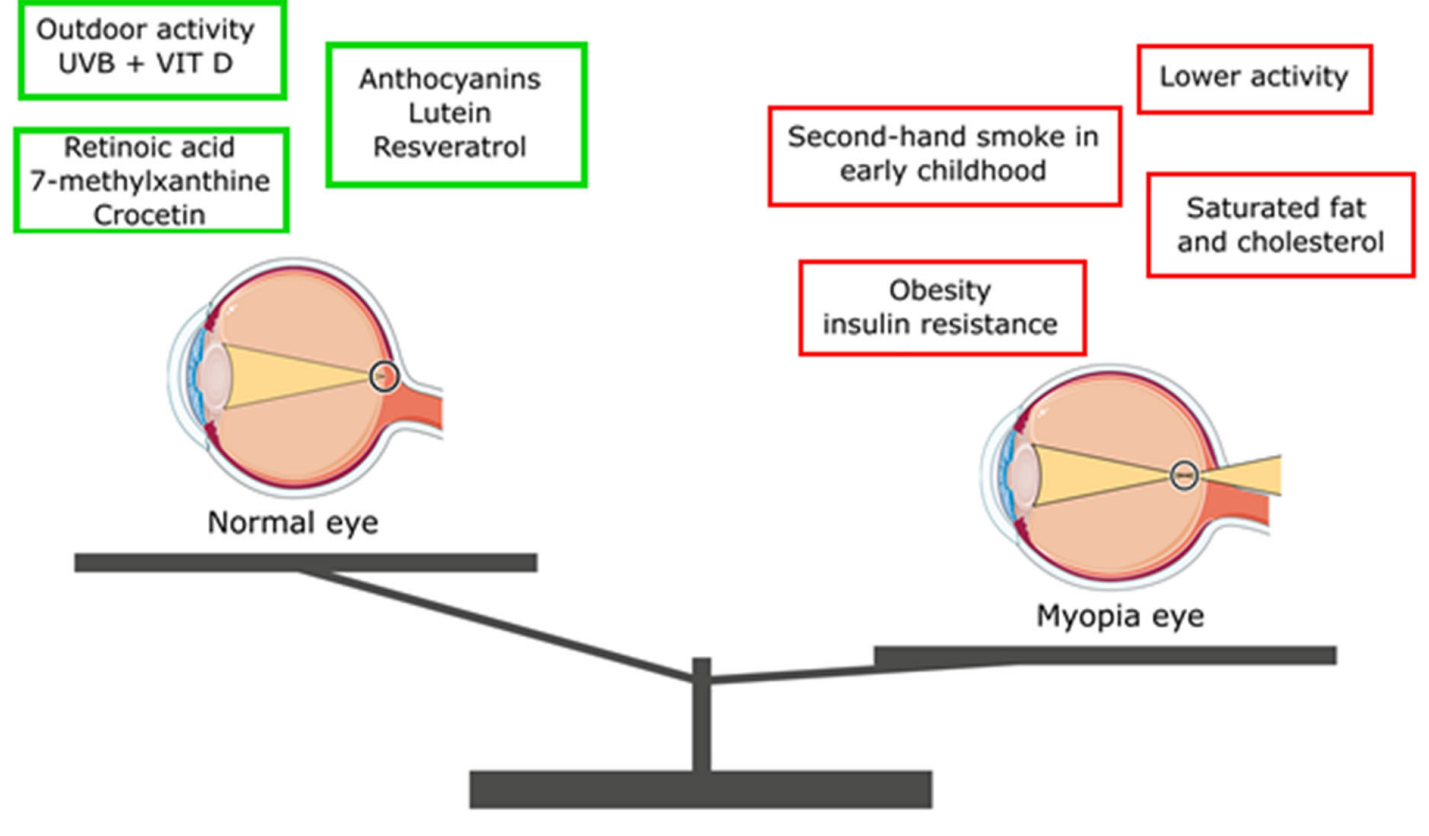
Possible factors that inhibit the risk of myopic development and progression (Normal eye—green frames) and possible factors that contribute to the development of myopia (Myopia eye—red frames). The figure was prepared based on the literature [31,89,91,100,101,102,103,104] and drawn by using pictures from Servier Medical Art. Servier Medical Art by Servier is licensed under a Creative Commons Attribution 3.0 Unported License (https://creativecommons.org/licenses/by/3.0/, accessed on 20 May 2023).

The scientific community holds differing opinions on the relationship between macronutrient consumption and the development and progression of myopia. One of the newest studies performed among Koreans proved that a greater dietary intake of carbohydrates, polyunsaturated and *n*-6 fatty acids, as well as vitamins and minerals, was associated with a reduced risk of myopia [87]. A study conducted in Hong Kong with 92 children revealed that those with lower total intakes of energy, protein, fat, and cholesterol at the age of 7 were more likely to develop myopia compared to their peers with normal dietary intakes [88]. Similarly, Lim et al. found that the higher consumption of saturated fat and cholesterol was associated with greater axial length of the eyeball. In a Singaporean study, the Singapore Cohort Study of Risk Factors for Myopia, which involved 851 children, it was observed that only the increased consumption of saturated fat and cholesterol was positively linked to axial eye length [89]. Berticat et al. discovered that higher carbohydrate intake increased the likelihood of myopia, but this effect was observed only in girls [90]. Conversely, Gardiner et al. reported that increasing the intake of animal protein in the diet of children with myopia resulted in a slower progression of the vision defect compared to a control group [91]. According to Kim et al., an elevated intake of carbohydrates, protein, phosphorus, iron, potassium, and sodium was connected to an increased risk of myopia, with excessive sodium intake being particularly linked to a 2.05-fold higher risk of developing the condition. Myopic children had a notably lower intake of fat, omega-3 fatty acids, and retinol but consumed more of other nutrients than their emmetropic and hyperopic peers [92]. Li et al. assessed the consumption of various food groups, including refined grains, sugar-sweetened beverages, protein foods, fruits, and vegetables, in 467 multi-ethnic children during their early years. They found no association between these dietary factors and the risk of myopia during the nine-year follow-up period [93]. It is worth noting that in Hong Kong, myopic children had lower protein and fat intake, which contrasts with observations in China, where myopic children had significantly higher protein intake [88]. In a study conducted with children in Singapore, none of these nutrients were associated with myopia [89]. The increased consumption of whole grains (>50%) was found to have a protective effect against myopia development. This happens because dietary supplements containing whole grains provide essential nutrients like calcium, iron, magnesium, manganese, copper, and zinc. Interestingly, changing the type of grains consumed (whole instead of refined) could be a potential public health approach to preventing myopia [94]. Pan et al. demonstrated a protective effect of omega-3 polyunsaturated fatty acids (ω-3 PUFA) against the development of myopia. Animal studies showed that daily ω-3 PUFA consumption (300 mg of docosahexaenoic acid [DHA] plus 60 mg of eicosapentaenoic acid [EPA]) reduced the development of myopia in guinea pigs and mice [95]. In humans, oral administration of ω-3 PUFA improved choroidal blood flow, reducing the risk of degenerative changes and scleral remodeling [95,96,97]. Increased dietary consumption of EPA (≥11 mg/1000 kcal) could be related to a reduced likelihood of high myopia in the group of patients aged 12–19. This happens because EPA promotes the relaxation of vascular smooth muscle cells and supports vasodilation [98]. Additionally, Liu et al. found that newborns fed breast milk rich in polyunsaturated fatty acids, such as arachidonic acid and docosahexaenoic acid, had a lower risk of developing myopia [99]. Figure 2 shows possible factors inhibiting the risk of myopic development and progression as well as the possible factors contributing to the development of myopia.

### 5.2. Micronutrients

**Figure 3:**
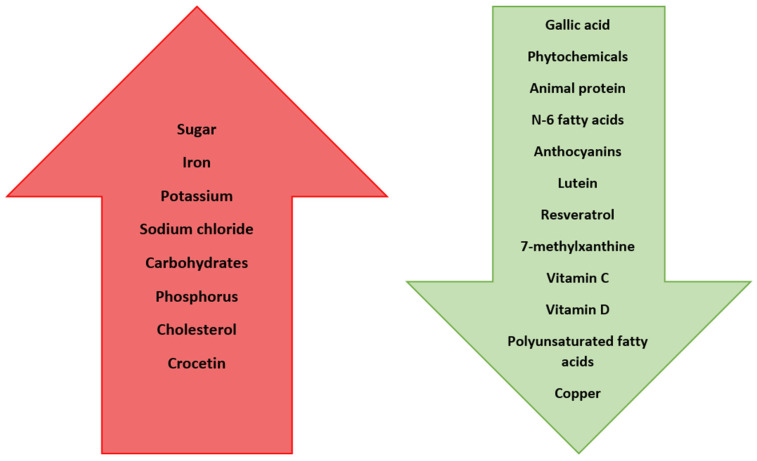
Diagram showing how nutrients affect eye development and myopia progression. Nutrients on the red arrow increase the risk of myopia and nutrients on the green arrow reduce the risk or progression.

Nakaishi et al. demonstrated that the oral consumption of blackcurrant anthocyanins effectively reduced apparent myopia induced by prolonged close work [105]. Similarly, anthocyanins derived from blueberry fruit have been found to have a relaxing effect on the ciliary muscle, a significant factor in myopia treatment [100]. Supplementation with lutein sourced from green leafy vegetables like kale and spinach enhances the functioning of photoreceptors in the retina, thereby improving visual acuity [106]. Lutein has also shown promise in inhibiting the development of myopia. Individuals with high plasma concentrations of lutein experienced a 40% reduction in the risk of myopia development [91]. Additionally, resveratrol (RSV) supplementation appears to hold potential benefits for myopia [101]. In a study involving hamsters treated with RSV, the axial length of their eyeballs was shorter compared to a control group. The RSV-treated eyes exhibited a decreased expression of myopia-related tissue remodeling proteins and inflammatory factors (such as, TGF-β, MMP2, TNFα, IL-6, and IL-1β), while the collagen I expression increased. These findings suggest that resveratrol might follow a similar regulatory pattern as atropine in myopia management [107]. Animal studies have also revealed the impact of substances like retinoic acid (a precursor to vitamin A), 7-methylxanthine (a caffeine metabolite), and crocetin (found in crocus and saffron) on the development of myopia [108]. The exact mechanisms of their actions remain unclear. It is theorized that 7-methylxanthine may control myopia progression by directly influencing scleral collagen content or by affecting muscarinic dopamine or acetylcholine receptors at the retinal level [109,110]. Mori et al. reported that the consumption of crocetin, a naturally occurring apocarotenoid dicarboxylic acid found in crocuses and saffron, has the potential to prevent myopia development [111,112] by improving choroidal circulation [108]. It is worth mentioning that a high intake of vitamin C turned out to be related with a decreased risk of myopia; the myopia prevalence was reduced by 38% (0.62-fold reduction) [113]. Among the other vitamins that play a role in eye function, vitamins D and A are frequently highlighted. Vitamin D is involved in regulating gene expression, the immune system, inflammation, cell proliferation and differentiation, apoptosis, and angiogenesis. Vitamin D3 (cholecalciferol) is synthesized from 7-dehydrocholesterol in the skin’s epidermal layer when exposed to sunlight, and it can also be obtained from dietary sources like eggs, dairy products, and fatty fish [102]. Given that the primary source of vitamin D is sunlight exposure, which has been shown to have a myopia-inhibiting effect, there is a belief that vitamin D could offer protection. Choi et al. discovered that individuals with higher serum levels of 25-hydroxy vitamin D (25(OH)D) had a lower incidence of myopia. Their findings revealed a significant association between spherical equivalent and serum 25(OH)D concentration [114]. Similar results were reported by Mutti et al. [115]. Tideman et al. established an inverse relationship between the serum 25(OH)D levels and eyeball length, with low levels increasing myopia risk [116]. These findings align with the results of other researchers [117,118,119]. Wahyudi et al. proposed that vitamin D supplementation and sunlight exposure could elevate serum 25(OH)D levels, potentially reducing myopia incidence and progression [120]. However, there are also studies in the literature where no link between vitamin D and myopia was demonstrated [121,122]. Vitamin A is vital for safeguarding against night blindness and preventing the thinning of the cornea, which can lead to corneal perforation and vision loss [123]. In fact, vitamin A deficiency is the leading cause of blindness in children in developing countries [124,125]. Ng et al. examined vitamin A intake through dietary questionnaires completed by 642 individuals at the ages of 14, 17, and 20, and compared these data with ophthalmological measurements taken at age 20. Surprisingly, they found no correlation between the quantitative vitamin A intake and refractive errors [126]. Zinc, which is involved in various processes including vitamin A metabolism and is highly concentrated in the retina and choroid, yielded inconsistent results regarding its influence on myopia development. Fedor et al., in their study involving children and adolescents, observed that the average serum zinc levels were significantly lower in those with myopia compared to a control group [127]. Similar findings were reported by other researchers [128,129,130]. Conversely, Burke et al. found no association between these factors [131]. It is worth noting that dietary zinc intake and serum zinc concentrations tend to be lower in vegetarians compared to non-vegetarians [132]. Moreover, research on young adults in India indicated a higher prevalence of myopia among vegetarians [133]. Additionally, children and adolescents with myopia have been found to exhibit lower copper levels compared to their counterparts without myopia. Cai et al., in their analysis of trace elements in students’ hair, observed an inverse correlation between copper levels and the degree of myopia [134]. These outcomes are consistent with the findings of other researchers [128,129,130]. The summary of all the effects of mentioned nutrients is shown in Table 1 and Figure 3.

## 6. Lifestyle and the Progression of Myopia

The amount of time spent outdoors has been found to influence the risk of developing and progressing myopia [103]. Findings from the Sydney Myopia Study underscored that engaging in over two hours of outdoor activities daily significantly decreased the risk of myopia [135]. Similar outcomes were echoed by other researchers [136,137]. Sherwin et al., by analyzing the available literature, determined that each subsequent hour spent outdoors per week reduced the chance of developing myopia by 2% [138]. Likewise, Xiong et al., in their study published in 2017, showed that extended time spent outdoors reduced the incidence of myopia, but had no effect on inhibiting the progression in children who were already myopic [103]. This study suggested that prophylaxis was important in children at familial risk of myopia. The reasons why the amount of time spent outdoors affects nearsightedness have not been fully explained. There have been attempts to explain them using several theories. Demand for accommodation, light intensity, or changes in the composition of chromatic light are the factors most often taken into account [35]. Williams et al. found that high UVB exposure was directly related to the time spent outdoors and exposure to sunlight and was associated with a reduced likelihood of nearsightedness. UVB exposure between the ages of 14 and 29 years was associated with the highest reduction in myopia odds in adults [31]. This phenomenon is explained by the activation of the light-stimulated dopaminergic amacrine cells of the retina, which may affect the axial growth of the eyeball [31,35], and higher concentrations of vitamin D in the serum induced by sunlight [31]. Notably, the animal models of myopia have verified that heightened light intensity can entirely impede the development of experimental myopia [35]. However, certain studies posit that the exposure to UVB and its dependent vitamin D pathway have no protective effect on the progression of myopia [116,139,140]. Improvements in lighting conditions, breaks from work, and looking at a distance have not been shown to slow down the progression of myopia. Also, the height of the table was not significant in the development and progression of the defect [137]. According to Liu et al., the effectiveness of physical activity should be seen as a strategy for improving the visual health of children and adolescents. Among the different forms of physical exercise, badminton and table tennis have emerged as the most advantageous for this goal. These activities involve quick transitions between distant and close-up vision, training the ciliary muscles to contract and relax rapidly, which enhances their sensitivity and accuracy [141]. Physical exercise alleviates the eye fatigue resulting from prolonged focus on stationary objects and enhances overall body health, promoting the development of visual function in teenagers and preventing the deterioration of myopia and other visual capabilities, as well as decreasing the likelihood of developing myopia in children aged six to twelve and enhancing eye health. The prevalence of myopia was consistently greater in females than in males across the various categories of physical activity [104]. Exposure to secondhand smoke in early childhood has been shown to slightly increase the risk of early-onset myopia. Chua et al. studied 197 children exposed to secondhand smoke from birth to 6 months of age. Children exposed to tobacco smoke during this period of life experienced more myopia compared to children without exposure. The risk of myopia in a child was even higher if the smoker indulged in smoking at home, in the car, or in the immediate presence of the child [142]. In the available literature, you can also find works assessing the impact of sleep on the development of myopia. However, the results obtained by the authors are different. Stafford-Bell et al. showed no relationship between the amount of sleep and changes in refractive error, axial length, or corneal curvature [143]. Huang et al., studying Chinese children, also found that sleep time was not related to myopia, the spherical equivalent (SE), and the axial length of the eyeball (AL) [144]. Similarly, the GUSTO study conducted in Singapore on 3-year-old children showed no relationship between sleep duration and myopia, SE, or AL [145]. On the other hand, Wei et al., studying Korean teenagers aged 12–19, found a negative correlation between sleep time and myopia [146]. Similar conclusions were drawn by other researchers [147,148]. Xu et al. found a lower risk of myopia in children who slept 9 h/day compared to those who slept 7 h or less/day [148]. Also, Ayaki et al. found that children with high myopia had the shortest sleep time [147]. The authors attribute this correlation to the relaxation of the ciliary muscle during sleep, which might impact myopia progression. Nonetheless, it is noteworthy that results concerning this correlation are somewhat disparate. Notably, myopia has not been linked to the duration spent watching TV or engaging in non-outdoor sports activities [148]. The key finding of the study conducted by Kim et al. revealed a strong link between myopia and age-related eye conditions, particularly cataracts and macular degeneration, in Korean adults aged 40 and older. Clinicians should be encouraged to incorporate early screening protocols for these conditions in patients with myopia. There was no such connection found between myopia and glaucoma [88].

## 7. Conclusions

Based on the literature reviewed, it can be concluded that there are no standardized, universally effective guidelines for reducing the risk of developing or advancing myopia. Given the increasing prevalence of myopia among children, further research in this area is undoubtedly warranted. Nonetheless, it remains crucial to advocate for a healthy lifestyle within society. In this era of pervasive multimedia exposure, promoting physical activity and outdoor time is of paramount importance. The varying rates of myopia progression between the sexes also necessitate further research to comprehend the mechanisms behind this phenomenon. It is very important to educate myopic patients about methods of preventing the development of myopia, as well as the factors that may have an adverse impact on the progression of vision defects. Also, children should be educated, and such activities could be carried out in schools. Parents of myopic children should also be aware of the importance of a proper diet and lifestyle. Additionally, ophthalmologists play a vital role in educating parents about potential approaches to myopia correction. Research has shown that relying solely on single vision glasses may not always be the optimal solution, particularly in cases where children experience a rapid progression of their visual impairment. Consequently, the alternative correction methods should be considered. Considering the emerging evidence highlighting the potential influence of specific nutrients and dietary components on myopic development, maintaining a well-balanced diet is also crucial.

## Figures and Tables

**Table 1:** A summary of the effects of specific nutrients on myopia progression and development.

Nutrient	Effect on Myopia Progression/Development
Gallic acid	Reduces the development of myopia by lowering oxidative stress
Sugar	Triggers the secretion of IGF-1, which influences cell growth and differentiation, leading to the development of myopia
Phytochemicals (e.g., carotenoids)	Prevent myopia development
Sodium chloride	Triggers fluid retention in the vitreous of the eye, which leads to myopia development
Iron	Elevated intake increases the risk of myopia
Potassium	Elevated intake increases the risk of myopia
Carbohydrates	Elevated intake increases the risk of myopia
Protein	Increasing the intake of animal protein slows down the progression of myopia; conclusions remain contradictory
Phosphorus	Elevated intake increases the risk of myopia
*N*-6 fatty acids	Greater intake reduces the risk of myopia
Cholesterol	Increased consumption increases the chance of developing myopia
Anthocyanins	Reduce apparent myopia
Lutein	Reduces the risk of myopia development by 40%
Resveratrol	Reduces the risk of myopia development by decreasing expression of TGF-β, MMP2, TNFα, IL-6, and IL-1β
7-methylxanthine	Helps to control myopia progression
Crocetin	Prevents myopia development by improving choroidal circulation
Vitamin A	No significant associations of its effect on myopia
Vitamin C	Reduces the development by 38%
Vitamin D	Reduces myopia incidence and progression
Polyunsaturated fatty acids	Have protective effect against the development of myopia
Copper	Deficiency predisposes patients to develop myopia
Zinc	Inconsistent results regarding its influence on myopia development

## Data Availability

All the materials and information will be available upon an email request to the corresponding author.

## Supplementary Materials

The following supporting information can be downloaded at: https://www.mdpi.com/article/10.3390/nu16193331/s1, PRISMA 2020 Checklist.
